# Comparative Evaluation of Retention of Fiber Posts in Different Dentin Regions Using Various Bonding Techniques: An In Vitro Study

**DOI:** 10.7759/cureus.33971

**Published:** 2023-01-19

**Authors:** Yashini Thanikachalam, Sadasiva Kadandale, Sangita Ilango, Revathy Parthasarathy, Sankar Vishwanath, Srividhya Srinivasan

**Affiliations:** 1 Conservative Dentistry and Endodontics, Chettinad Dental College and Research Institute, Chennai, IND

**Keywords:** total-etch, retention, glass fiber post, dentin, bonding agent

## Abstract

Aim

This study aims to evaluate the retention of fiber posts in the coronal, middle, and apical dentin regions with various bonding techniques by using fifth, sixth, and eighth-generation bonding agents and self-etch adhesive cement.

Materials and methods

For this study, 96 freshly extracted human incisors with straight roots were selected. Endodontic treatment of the specimens was performed. The post spaces were created immediately after obturation and the posts were luted with three different bonding agents and self-adhesive resin cement and the specimens were divided into four groups. Approximately 3 mm thick sections were made from different dentin regions of the post space and were tested for three subgroups: Subgroup I: Coronal, Subgroup II: Middle, Subgroup III: Apical. The specimens were tested on a universal testing machine.

Results

Statistical analysis was done using one-way analysis of variance (ANOVA) and Tukey’s post hoc test using SPSS software version 17.0 (SPSS Inc., Chicago ). The intra-group comparison showed that the bond strength was more in the fifth-generation bonding agent (Cervical- 8.2574± 1.49034, Middle- 11.4800± 2.59938, Apical- 14.7108±1.82931), followed by the sixth (Cervical- 9.102± 2.76119, Middle-9.3152±2.11585, Apical- 12.9478±4.69404) and eighth-generation bonding systems (Cervical- 9.0938±2.77537, Middle- 7.1585±1.97601, Apical- 9.3726±0.73720) and the self-etch adhesive dental resin cement (Cervical- 5.1004±2.17389, Middle- 4.1574±1.28664, Apical-7.8884±1.90078). The inter-group comparison showed that the bond strength was higher in the fifth-generation bonding agent followed by sixth-generation, eighth-generation, and self-adhesive resin cement.

Conclusion

The present study reveals that the highest push-out bond strengths were obtained in apical sections followed by the middle and cervical areas. The bond strength was higher when using the fifth-generation bonding agent followed by the sixth, eighth, and self-adhesive resin cement. Despite many advancements, the fifth-generation bonding agent still showed superior retention in different dentin regions among various other bonding techniques.

## Introduction

Restoration of endodontically treated teeth is one of the most common challenges encountered by dentists worldwide. When there is excessive loss of coronal tooth structure, posts and cores are the preferred choice of treatment in such teeth and retention for the final coronal restoration is provided by the cementation of the post within the root canal [1].

Fiber-reinforced posts introduced in the 1990s are capable of bonding to resin cement and can transport the stresses generated between the root canal and the post [2]. Their unique properties, like high tensile strength, fatigue resistance, and stiffness (modulus of elasticity), like that of dentin, result in fewer root fractures [3]. Initially, their use was limited due to difficulties in achieving bonding to intracanal dentin, which was overcome by using dentin bonding adhesives along with a resin cement of similar flexibility, which provides improved adhesion within the root canal space [4].

The fifth-generation adhesives were introduced in the 1990s as a one-bottle system with combined primer and adhesives to simplify the working process, which resulted in less working time and minimal postoperative sensitivity [5]. The sixth-generation adhesives or the self-etch adhesives eliminated the need for etching and are less dependent on the dentin’s hydration state [6]. Eighth-generation bonding agents were nano-filler adhesives with a higher bond strength and the ability for fluoride release [7]. More recently, self-etch adhesive cement was introduced to enable a time-saving direct application of the luting material, eliminating the need for dentin adhesives. In this study, fiber-reinforced posts were tested with various dentin bonding agents and self-etch adhesive cement to assess the bond strength in different dentinal areas.

The aim of this study was to evaluate the retention of fiber posts in the coronal, middle, and apical dentin regions by using fifth, sixth, and eighth-generation bonding agents and self-etch adhesive cement. The null hypothesis was that there is no significant difference in retention among these bonding agents in different dentin regions.

## Materials and methods

Ninety-six human maxillary central incisors that were extracted for periodontal reasons were used in this study. Teeth with straight roots and root lengths of 16 mm were selected for this study. Teeth having curved roots, root caries, root resorption, root fractures, and root length less than 16 mm were excluded from this study. The selected teeth were stored in normal saline at room temperature until use, disinfected with sodium hypochlorite solution, and cleaned with an ultrasonic scaler. The crowns were sectioned using a double-faced diamond disc at the level of the cementoenamel junction (CEJ) at a low speed to facilitate instrumentation.

The canal patency was achieved with a #10 k file followed by #15 and #20 files, and the working length was determined to be 15 mm. All the root canals were instrumented by using K-files (Dentsply Maillefer, Ballaigues, Switzerland). The biomechanical preparation was done by the crown down technique with XsmartTM plus the Endo Motor (Dentsply Maillefer) and Protaper Gold files (Dentsply Sirona) till F3. Five ml of 5% solution of sodium hypochlorite and 17% ethylenediaminetetraacetic acid (EDTA) solution were alternatively used for irrigating the canals.

After preparation, the canals were blotted with absorbent paper points (Endo MAX, Dento One Inc., Texas). The root canals were obturated with AH sealer (Dentsply Maillefer) and F3 Gutta-percha points (Dentsply Maillefer) using the cold lateral condensation technique.

Immediately after obturation, the post-space preparation was completed up to a #4 Peeso Reamer (Dentsply Maillefer) leaving 3-5 mm of Gutta-percha in the apical region. 5 ml of 5% sodium hypochlorite was used during and after preparation of post space to irrigate the canals. After post-space preparation, 17% EDTA was used to irrigate the canals. Again 5% sodium hypochlorite solution was used for final irrigation. The fiberglass posts (Angelus, Brazil) were inserted into the post space, tested for fit, and sectioned 2 mm above the coronal margin of the specimen with a double-faced diamond disc.

The specimens were divided into four groups of 24 teeth each: Group I - 5th generation dentin bonding agent ((Tetric n-bond total-etch (Ivoclar Vivadent)); Group II - 6th generation dentin bonding agent (Multilink n primer a and b (Ivoclar Vivadent)); Group III - 8th generation dentin bonding agent (Tetric n-bond universal (Ivoclar Vivadent)); Group IV - Self-adhesive resin cement. (SpeedCEM Plus (Ivoclar Vivadent)).

In groups I, II, and III, after acid etching, the bonding agent was applied as per the manufacturer’s instructions and light-cured for 20 seconds followed by the application of RelyX™ (3M ESPE products, St. Paul, MN) for luting the posts inside the prepared post space. The excess was removed and light-curing was done from the coronal ends of the posts for 20 seconds with a light-emitting diode (LED) light-curing unit. In group IV, the resin cement was applied directly onto the surface of the posts, which were then inserted into the space prepared and light-cured for 20 seconds. All the samples were then stored in water at room temperature for 24 hours.

Each specimen was sectioned at right angles to the long axis to obtain 3mm thick slices. One slice from the coronal, middle, and apical regions of the post space from each group was obtained and divided into three subgroups, as follows: Subgroup 1: Coronal; Subgroup 2: Middle; Subgroup 3: Apical.

Estimation of push-out bond strength

**Figure 1:**
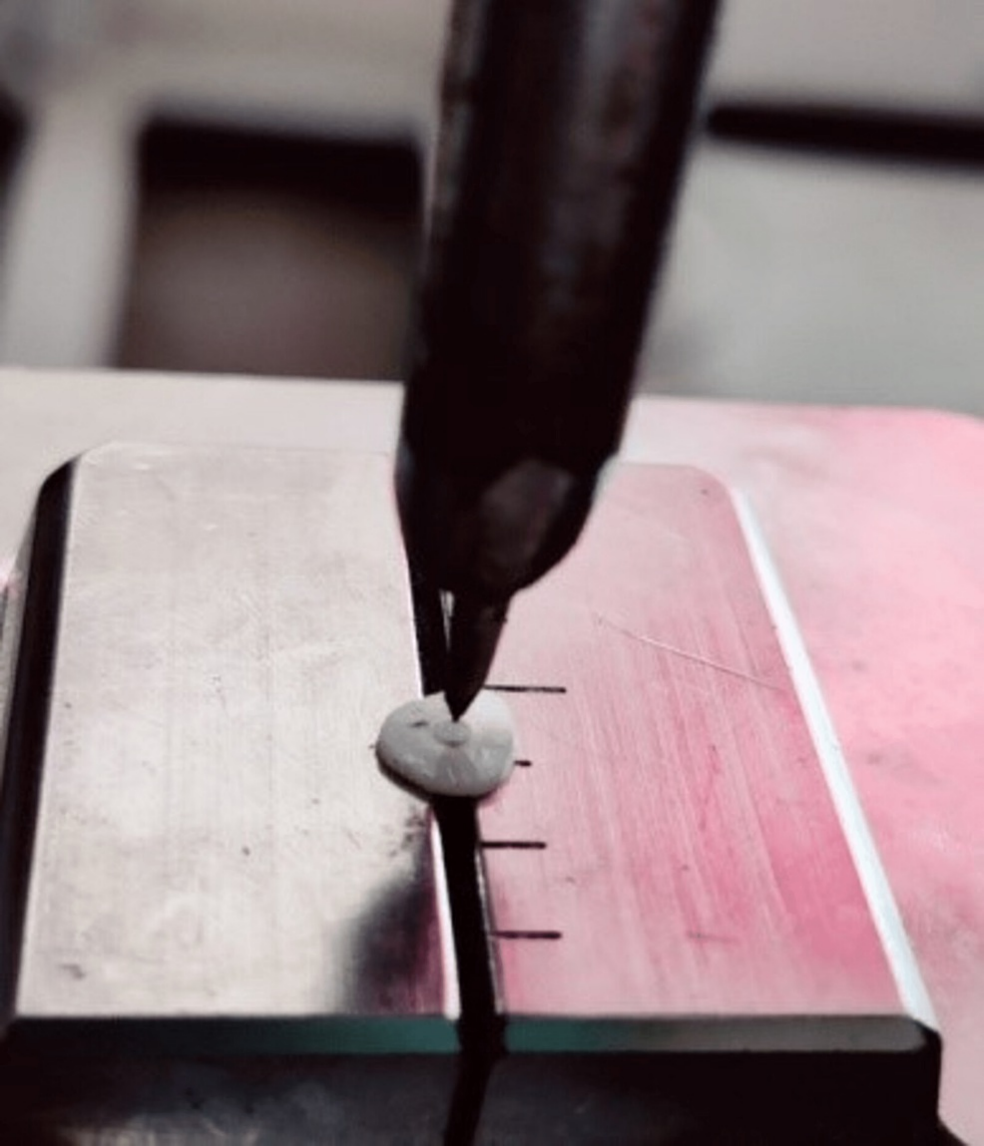
Tip of the Universal Testing Machine directed to the center of the fiber post

The slices from the test specimen of four different groups were fitted to the Universal Testing Machine (Shimadzu AG-Xplus50KN, Japan). The tip of 0.8 mm diameter was placed over the test specimen and the application of compression force was in an apical-coronal direction until the post piece was dislocated at a cross-head speed of 0.5 mm/minute (Figure 1). The peak value of load that was required for post-dislocation from each sample was recorded in Newtons. The push-out bond strength (measured in megapascals) was measured by dividing the recorded peak value of load by the bonding surface area, i.e., push-out bond strength (MPa) = maximum load (N)/bonding surface area (mm^2^).

Statistical analysis

The intra-group and inter-group comparisons were done by one-way analysis of variance (ANOVA), and multiple pairwise comparisons were done with Tukey’s post hoc test. Statistical significance was accepted at a level of P<0.05. Data were analyzed using SPSS software version 17.0 (SPSS Inc., Chicago).

## Results

There is a significant difference in push-out bond strength in Group I among the three sites wherein bond strength is significantly greater in the apical region, followed by the middle and coronal regions as seen in Table 1.

**Table 1:** Intra-group comparisons of push-out bond strength

Groups	Areas	Mean	SD	95% CI		F	P value
Group I	Coronal	8.2574	1.49034	6.4069	10.1079	12.8	0.001
	Middle	11.4800	2.59938	8.2524	14.7076		
	Apical	14.7108	1.82931	12.4394	16.9822		
Group II	Coronal	9.1020	2.76119	5.6735	12.5305	2.05	0.17
	Middle	9.3152	2.11585	6.6880	11.9424		
	Apical	12.9478	4.69404	7.1194	18.7762		
Group III	Coronal	9.0938	2.77537	5.6477	12.5399	1.79	0.20
	Middle	7.1586	1.97601	4.7051	9.6121		
	Apical	9.3726	.73720	8.4572	10.2880		
Group IV	Coronal	5.1004	2.17389	2.4012	7.7996	5.67	0.019
	Middle	4.1574	1.28664	2.5598	5.7550		
	Apical	7.8884	1.90078	5.5283	10.2485		

There is no significant difference in push-out bond strength in Groups II and III among the three sites. There is a significant difference in push-out bond strength in Group IV among the three sites where bond strength is significantly greater in the apical region, followed by the coronal and middle root regions as seen in Table 2.

**Table 2:** Inter-group comparison of push-out bond strength at three dentin sites

Site	Group	Mean	SD	95% CI		P-VALUE
Coronal	I	8.25494	1.498169	6.39472	10.11516	0.050
	II	9.10282	2.766907	5.66725	12.53839	
	III	9.09462	2.776987	5.64653	12.54271	
	IV	5.09712	2.172521	2.39958	7.79466	
Middle	I	11.48738	2.606364	8.25115	14.72361	0.001
	II	9.30782	2.112778	6.68446	11.93118	
	III	7.16352	1.975920	4.71009	9.61695	
	IV	4.16314	1.279590	2.57432	5.75196	
Apical	I	14.70424	1.832254	12.42920	16.97928	0.004
	II	12.94698	4.695880	7.11628	18.77768	
	III	9.37834	.744743	8.45362	10.30306	
	IV	7.89332	1.901148	5.53273	10.25391	

The post hoc test shows that there is a significant difference in push-out bond strength in the middle root and apical root sites. There is no significant difference in push-out bond strength between the groups in the coronal region. In the middle root region, the push-out bond strength was significantly different between Group I and Group IV, Group I and Group III, and Group II and Group IV. In the apical root region, the push-out bond strength was significantly different between Group I and Group IV & Group I and Group III.

## Discussion

In the present study, the push-out bond strengths of three different bonding agents and one self-etch adhesive cement in three different dentin regions are measured and the values are expressed in megapascal (MPa). Cementation is a crucial step for the placement of any post inside the root canal to ensure the longevity of the core build-up material, thereby maximizing the longevity of the tooth.

The factors that can affect the retention of posts are post shape (conical/cylindrical), post dimensions (length and diameter), intracanal space preparation, type of surface (serrated/screw/smooth), operator skills, and the type of cement used [8]. The retention of posts can be increased by roughening the prepared surface of dentin by increasing the surface area and by enhancing the mechanical interlocking that is present between the surface of the dentin and the cement [9]. The major morphological difference present between the bonds of self-etch and total-etch is the thickness of the hybrid layer [10]. Though the hybrid layer created by the self-etch systems is not thicker than total-etch systems, bond strength comparison between the two has controversial results [10]. In a study by Domnez et al., it was found that the total-etch bonding agent showed up to 8 μm formation of a hybrid layer and resin tags measuring 5 μm to 10 μm. In the self-etching sixth-generation bonding agent, the hybrid layer was about 2 μm to 3 μm and resin tags measuring about 5 μm to 7 μm were seen. Total-etch adhesive systems and self-etching adhesives [11] are more likely to have discrepancies between the depth of infiltration and the extent of demineralization of the adhesive resin.

Translucent fiber posts have high tensile strength and fatigue resistance and their modulus of elasticity is like that of dentin [10]. These posts must allow light to transmit into the canal and would increase the degree of conversion of dual-cure resin along with the improvement of mechanical properties like hardness and modulus of elasticity. Therefore, it is recommended to use self-curing or dual-cured composites for luting fiber posts [12]. According to the results of this study, the intra-group comparison showed that the bond strength was more in fifth-generation bonding agents, followed by the sixth and eighth-generation bonding systems and self-etch adhesive dental resin cement.

In Group I, the greatest retention mean values were observed for the apical third (14.7108±1.82931 MPa), followed by the middle third (11.4800±2.59938 MPa) and the coronal third (8.2574± 1.49034 MPa) for the subgroups investigated. Greater retention values seen in the apical third can be due to more intimate contact of the fiber post with the dentinal walls of the canal, mainly in the apical thirds that can form locking areas. Thus, retention of the post with root dentin walls in these regions occurs by mechanical overlapping and tenso-friction and by adhesive bonding [12].

In Group II, there is no statistically significant difference in the push-out bond strength among the three sites (Coronal- 9.102±2.76119, Middle- 9.3152±2.11585, Apical- 12.9478±4.). As this generation has two bottles (Prime A and B), it is advised that the components should be mixed only before use. This mixture of hydrophobic and hydrophilic resin components is applied to the root [5]. It etches the dentin less aggressively than total-etch products and thus the advantage of this generation is that their efficacy is less dependent on the dentin’s hydration state when compared to that of fifth-generation bonding agents [6].

In Group III, there is no statistically significant difference in the push-out bond strength in among the three sites (Coronal- 9.0938±2.77537, Middle- 7.1585±1.97601, Apical- 9.3726±0.73720). It was estimated that the eighth-generation bonding agent is based on dual curing and nanotechnology. The silicon-dioxide nano-sized fillers (below 20 nm) that are present in eighth-generation agents are chemically cured while light curing is done. This can lead to cross-links that are stronger with hydrophilic and acidic components inside the smear layer and even claim that they can release fluorides [13]. In Group IV, the bond strength is significantly greater in the apical region, followed by the coronal region and middle root regions. The low push-out bond strength values of self-adhesive cement confirmed that they interact only superficially with the tooth structure and do not dissolve the smear layer [14]. Therefore, for the dental adhesion of the resin components, chemical bonding is responsible rather than micromechanical bonding.

A study by Alizadeh Oskoee et al. concluded that with the use of different adhesive systems, the bond strength of total-etch systems is greater than that of one- and two-step self-etch systems. They stated that by the use of phosphoric acid and etchants in etch and rinse systems, the smear layer is dissolved completely creating a path for the hybridization of decalcified intertubular dentin and dentinal tubule walls [15]. Self-etch primers have weak etching ability and they cannot eliminate the smear layer and can only partial penetration through the smear layer may be seen [16].

Tukey’s post hoc test for multiple pairwise comparisons showed that there is no significant difference in push-out bond strength between the groups in the cervical region. In the middle root region, the push-out bond strength was significantly different between Group I and Group IV, Group I and Group III, and Group II and Group IV. In the apical root region, the push-out bond strength was significantly different between Group I and Group IV & Group I and Group III.

Ashraf Elsayed Nasr et al. concluded that total-etching adhesive revealed higher micro-shear bond strength than self-etch adhesive regardless of the type of dentin tested; besides, the etch and rinse step improves the micro-shear bond strength of self-etch adhesive with a different type of dentin [17].

In the present study, based on the values of retention in the apical region, it is clear that the adhesive was polymerized effectively, regardless of the distance from the light source. According to the manufacturer, the fiber post used in this study has glass fibers that can conduct light energy, which might have helped in the polymerization of the adhesive system in the apical thirds of the canal [18]. The adhesive strength of root dentin could be related more to the surface of solid dentin than that of the density of dentinal tubules. Hence, there will be an increased push-out bond strength in the apical region, as the density of tubules decreases from the coronal to the apical third [19].

In contrast to the apical third, there will be a higher requirement in the volume of cementing material and increased stress at the adhesive interface while polymerization shrinkage occurs. This increase in polymerization shrinkage will be critical for adhesion with a high C-factor of the root canal [19]. Since the cement forms the weakest link between the root canal dentin and the post, the usage of a greater volume of cement can cause reduced push-out bond strength in the coronal third.

Many studies have been conducted to test the shear bond strength of different dentin bonding agents, but only sparse literature is available on testing the tensile bond strength of different bonding agents [13,20]. Moreover, recent studies comparing various bonding agents have shown that eighth-generation bonding agents have better bond strength than sixth and seventh-generation agents due to the presence of unique monomers that have the ability for calcium chelation that aids in chemical bonding to the tooth.

The type and way nanofillers in the eighth-generation adhesives are incorporated into the structure affect the viscosity of the adhesive and the ability of resin monomers to penetrate collagen fiber cavities. With dimensions greater than 15-20 nm or with content greater than 1.0% by weight, these systems can increase the viscosity of adhesives. It can also result in the clustering of fillers on the moist surface. These clusters can cause cracks and a decrease in binding force [21]. This may be the reason why eighth-generation bonding agents have shown decreased push-out bond strength in the present study.

In the present study, the fifth-generation bonding agent used (Tetric N bond) has shown higher push-out bond strength than other generations. One important reason for this result is the higher depth of resin tag formation due to its etch and rinse system. According to Hegde et al., in no-etch or self-etch adhesives, there is interference in micromechanical bonding due to the incorporation of calcium salts and dissolved proteins at the bonding interface [22].

The smear layer formed on the canal walls is thicker in nature when rotary post drills are used as compared to routine cleaning and shaping of the root canal. Thus, the use of phosphoric acid etching within the root canal aids in the dissolution of this thicker smear layer and allows more effective micromechanical retention of the resin cement for post luting. Bitter et al., in 2017, concluded that intracanal acid etching enhances the penetration capacity of dentin adhesives using confocal laser scanning microscopy (CLSM) analysis [23].

In fifth-generation dentin bonding agents, the phosphoric acid used promotes dentinal demineralization, thick hybrid layer formation, and higher adhesive impregnation into dentin. The hydrophobic adhesive component and hydrophilic primer components are chemically balanced and present in one bottle. The adhesive system contains water and ethanol as solvents, which play an important role in enhanced substrate bond strength, as they provide an increased wettability of acid-prepared dentin. The use of fifth-generation bonding agents also creates a hydrophobic layer above the dentin-bond interface that is responsible for its reduced water sorption. Partial hydroxyapatite dissolution occurs when fifth-generation adhesives are used, which is an important phenomenon that decreases collagen fiber degradation that ultimately improves the bond strength [24].

Retention of fiber posts was found to be higher when the adhesives were used in the total-etch mode than the self-etch mode. Ubaldini et al., in 2018, concluded that acid etching pre-treatment positively affected the retention of fiber posts when compared to self-etch adhesives and self-adhesive cement [25].

A recent study by Ishwarya et al. compared the bond strength and mode of failure of glass fiber posts luted using two different adhesives and concluded that superior bond strength was seen in two-step etch and rinse adhesives rather than self-etch adhesives due to enhanced resin cement penetration into the dentinal substrate [26].

These results are in accordance with the results obtained in the present study.

Proper selection of dental adhesives and luting agents for the improved retention of fiber posts in endodontically treated teeth is necessary for its long-term prognosis. More in vivo studies and in vitro studies with larger sample sizes are required to provide better insight and to evaluate the clinical success of these materials.

## Conclusions

Within the limitations of the current study, it can be concluded that the intra-group comparison showed that the pushout bond strength was more in fifth-generation bonding agents followed by the sixth and eighth-generation bonding systems and self-etch adhesive dental resin cement. The highest pushout bond strengths were obtained in the apical region followed by middle and coronal regions.
